# How useful is satellite positioning system (GPS) to track gait parameters? A review

**DOI:** 10.1186/1743-0003-2-28

**Published:** 2005-09-02

**Authors:** Philippe Terrier, Yves Schutz

**Affiliations:** 1 Department of Physiology, University of Lausanne, Switzerland

## Abstract

Over the last century, numerous techniques have been developed to analyze the movement of humans while walking and running. The combined use of kinematics and kinetics methods, mainly based on high speed video analysis and forceplate, have permitted a comprehensive description of locomotion process in terms of energetics and biomechanics. While the different phases of a single gait cycle are well understood, there is an increasing interest to know how the neuro-motor system controls gait form stride to stride. Indeed, it was observed that neurodegenerative diseases and aging could impact gait stability and gait parameters steadiness. From both clinical and fundamental research perspectives, there is therefore a need to develop techniques to accurately track gait parameters stride-by-stride over a long period with minimal constraints to patients. In this context, high accuracy satellite positioning can provide an alternative tool to monitor outdoor walking. Indeed, the high-end GPS receivers provide centimeter accuracy positioning with 5–20 Hz sampling rate: this allows the stride-by-stride assessment of a number of basic gait parameters – such as walking speed, step length and step frequency – that can be tracked over several thousand consecutive strides in free-living conditions. Furthermore, long-range correlations and fractal-like pattern was observed in those time series. As compared to other classical methods, GPS seems a promising technology in the field of gait variability analysis. However, relative high complexity and expensiveness – combined with a usability which requires further improvement – remain obstacles to the full development of the GPS technology in human applications.

## 

Analysis of the pattern in cyclic movements may be of great interest in neurosciences and behavioral sciences, since they rely on complex sensory-motor coordination requiring both automated and voluntary tasks [1]. Recent studies, based on non-linear analysis of time series, have shown the presence of complex temporal fluctuations in several biological repetitive processes, such as heart beats [2-4], respiration [5], or controlled finger movements [6].

Walking is the one of the most common repetitive movement that humans performed in real life. In addition to automatic rhythmic activation by Central Pattern Generators at the spinal level, the locomotor system is regulated by the cerebellum, the motor cortex and the basal ganglia, with feedback from proprioceptive, visual and vestibular sensors. Stride after stride, the final output of the control segment modulates the spatial (Step Length, SL), and temporal (Step Frequency SF or cadence) patterns of the gait in order to provide optimal movement in terms of mechanics and energetics [7-11].

Gait variability can be defined as the variation of gait parameters from stride to stride. It was reported that gait variability could by modified by different pathology (e.g. neuro-degenerative diseases), or to be related to the propensity to fall in elderly [12,13]. In addition, it has been shown that stride-to-stride variability diminished with the maturation of the gait in children [14].

Hausdorff's group has extensively studied long-term gait variability [12-21]. They reported [20] that the stride-to-stride variation of stride duration exhibited long-range, self-similar correlations. In other words, the fluctuation in the stride interval is characterized by an autocorrelation function that decays as a power law: the present value is statistically correlated not only with its most recent value but also with its long-term history in a scale invariant fractal manner [20,21]. They attempted to demonstrate the implication of basal ganglia in the control of the stability and the generation of the fractal pattern. In short, the underlying hypothesis is that fractal pattern is a marker for neural complexity: different factors (disease, aging, imposed stride frequency by metronome, called metronome walking) that affect this complexity lead to the loss of fractal patterns and to the emergence of random patterns [15].

For all these different experiments, Hausdorff et al. used a force-sensitive switch placed in shoes [17]. This sensor detects heel strike and therefore allows to obtain information about temporal pattern of the gait only. They addressed the issue as follows: "Additional information regarding the alterations of gait [...] might be provided [...] by obtaining stride-by-stride measures of stride length and gait speed" [18].

In this context, we propose the use of high-accuracy satellite positioning (Global Positioning System, GPS), as a alternative tool to obtain long time series of basic gait parameters, i.e. Walking Speed (WS), Step Length (SL) and Step Frequency (SF). The purpose of the present review article is to highlight the new GPS technique and compare it to other gait analysis methods. We present a thorough description of theoretical and practical aspects of GPS technology for high accuracy positioning. Next, we describe the underlying biomechanical assumptions necessary to obtain gait parameters from GPS positioning data. Finally, following a discussion of our recently published results about fluctuation analysis of gait parameters [22], we highlight the advantages and shortcomings of GPS techniques as compared to other methods.

## Motion analysis: classical methods

**Figure 1:**
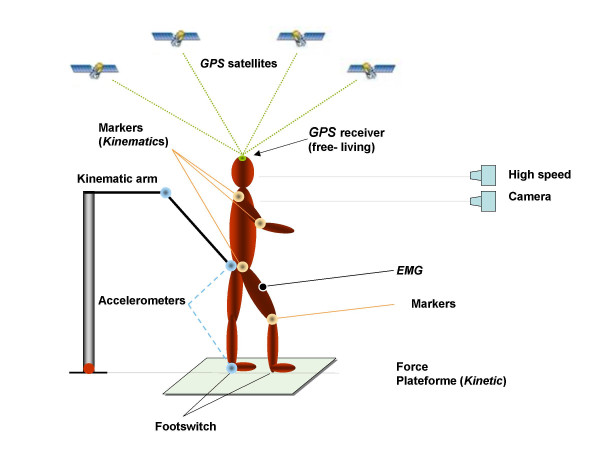
*Simplified scheme of the techniques available for gait analysis*. Each method measure different parameters and have different advantages and shortcomings.

Several gait analysis techniques have been developed over the last decades (fig. 1) [23]. A *kinematic *analysis of gait requires measurement of the displacement of the body segments during the walking cycle. Electrical, photographic, cinefilm and video or other electronic techniques have been used to calculate the position and orientation of each body segment to reconstruct the movements that took place. Measurement can be made in two or three dimensions. In order to understand how walking is accomplished, the forces acting on the human body must be also assessed (*kinetics*) [8,9,24,25]. By analyzing the moments and forces occurring at the joints to produce the motions of the limbs, an estimation can be made of the forces the muscles must produce. For a complete kinetic analysis of each body segment, kinematic data (displacements, velocity), anthropometric data (body segment parameters), and external force data (gravity, ground reaction force) are required. The ground reaction force is classically measured by a force plateform [25,10]. This device determines the magnitude and direction of the ground reaction force vector by measuring its three components (vertical, mediolateral and anteroposterior shear forces) and vectorally adding them. In parallel, in order to evaluate muscle activity, the depolarization of the muscles membrane by motor neuron activation can be tracked by using Electromyography (EMG).

While a number of gait analysis systems have been developed over the years to allow an accurate and overall description of walking, most of them are impractical for fast-paced clinical settings. Furthermore, they are not designed to record long times series of gait parameters over numerous consecutive strides. Alternative techniques have been therefore used in order to analyze a reduced set of parameters with an increased practicability. Instrumented walkway [26] permits a rapid survey of several temporal and spatial gait parameters (step length, step width, stance/swing time, step duration, etc.); however, the distance is limited (typically 10 meters), and the subject must follow a straight trajectory.

The shortcoming of limited space in a laboratory environment can be partially overcome by using a treadmill. Video analysis or instrumented treadmill (force plateform [27] or kinematic arm [28-30]) allow investigators to analyze long duration walking or running. In theory, treadmill walking is supposed to be energetically and biomechanically identical to normal walking. However, treadmill walking alters the perception of motion by the participant and therefore may alter the gait parameters as compared to free walking. In addition, because of the narrow path offered by the treadmill, there is no freedom in the selection of the trajectory.

In parallel, other methods – based on portable sensors – have been developed to increase usability of gait analysis under free walking conditions. Accelerometers and gyroscopes have been used to retrieve several temporal and spatial gait parameters [31-37]. These techniques are very promising, however they rely on complex algorithms to convert raw measurements (acceleration, angular motions) into gait parameters (speed, step length, cadence). In addition, these algorithms are mostly calibrated to normal walking under standard conditions: there is no warranty that environmental changes (slope, quality of the terrain) or pathological gait (for instance claudication) are correctly taken into account. As a result, investigators must carefully select their devices and extensively test whether they obtain an output compatible with their experimental conditions. In our opinion, a less indirect methodology would offer more flexibility in the experimental design; by allowing a direct speed and position measurement, GPS is a good candidate for such an approach.

In 1995, Hausdorff and colleagues proposed a new footswitch method to analyze long term variability of the gait [17]. With a small portable sensor in the shoe, it is possible to retrieve stride duration stride by stride over very long periods (1 hour walking, [21].). However, it is not possible to assess spatial parameters (SL) by using this technique.

## GPS in human applications: historical perspectives

Almost ten years ago, we proposed to utilize GPS for assessing physical activity in free living conditions, in particular walking and running [38]. Simple relatively cheap commercial instruments used for leisure navigation (e.g. sailing) was tested. Using this type of GPS receiver, it was concluded that the accuracy of speed was insufficient for research purpose and that it could be improved by using differential GPS (DGPS). In a subsequent study, it was shown that DGPS improved the speed accuracy by a factor of about 10 as compared to non-differential GPS (error below 0.1 km/h) [39]. However, the study was performed when the satellite signals was voluntarily degraded by the US Departement of Defense (Selective Availability), so that the improvement with DGPS is expected to be considerably greater than today (since SA was removed in 2000). Witte & Wilson [40] have shown, using non-differential GPS, that reasonable accuracy in straight trajectory could be observed, but the error increased in circular path especially with small radii of curvature where a tendency was observed to underestimate speed [40]. More recently, another group in Scandinavia used DGPS for assessing the performance of orienteering with DGPS, and suggested that it could be combined with complementary techniques (accelerometry, electromyography etc.) in the field of outdoor exercise physiology [41-43].

## Standard GPS: principles

The Global Positioning System (GPS) is a satellite-based navigation system made up of a network of 24 satellites placed into orbit by the U.S. GPS works in any weather conditions, anywhere in the world, 24 hours a day. There are no subscription fees or setup charges to use GPS. GPS satellites circle the earth in a very precise orbit and transmit signal information. GPS receivers make use of triangulation to calculate the user's exact location. Essentially, the GPS receiver compares the time a signal was transmitted by a satellite with the time it was received. The time difference tells the GPS receiver how far away the satellite is. With distance measurements from a few more satellites, the receiver can determine the user's position.

GPS satellites transmit two low power radio signals, designated L1 and L2. The signals travel by line of sight, meaning they will pass through clouds, glass and plastic but will not go through most solid objects such as buildings and mountains.

A GPS signal contains three different bits of information – a pseudorandom code, ephemeris data and almanac data. The pseudorandom code is simply an I.D. code that identifies which satellite is transmitting information. Ephemeris data contains important information about the status of the satellite (healthy or unhealthy), current date and time. This part of the signal is essential for determining a position. The almanac data tells the GPS receiver where each GPS satellite should be at any time throughout the day. Each satellite transmits almanac data showing the orbital information for that satellite and for every other satellite in the system.

## High accuracy GPS: principles

**Figure 2:**
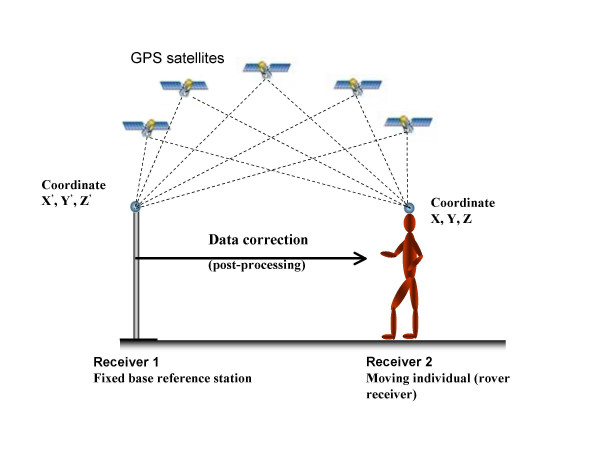
*Differential GPS principles*. The satellites are viewed by both receivers, located closed to each other. Reference receiver 1 calculates signal errors for GPS satellites. The correction is used to enhance navigation accuracy of receiver 2.

Assuming that two GPS receivers are close to each other (0–50 km), the different errors reducing the positioning accuracy (mainly atmospheric disturbance) affect both receivers the same way and with the same magnitude. If the exact location of one receiver is known (base receiver), this information can be used to calculate errors in the measurement and then report these errors (or correction values) to the other receiver with unknown position (rover receiver), so that it could compensate for them. This technique is called differential mode (DGPS, see fig. 2). This differential mode removes almost all errors except multipath (fake reflected signals) and receiver errors, because they are local to each receiver. The receiver error is typically about 10 cm for standard DGPS (differential code). If range errors are transmitted from the base receiver to the rover in real-time (radio link), then the system is called real-time DGPS. If real time results are not needed (typically in biomechanics), the measurement are time tagged and recorded in the base and rover receivers and later transferred to a computer to correct the data and calculate an accurate position of the rover at each instant (post processed DGPS).

Real Time Kinematics (RTK) is based on measuring distances to the satellites with carrier phase. As DGPS, this mode requires two receivers (base and rover), but the positioning does not rely on the pseudorandom code sent by satellites, which directly allows the estimation of the distance between the receiver and each satellite. Instead, the electromagnetic carrier of the signal is compared to a similar wave generated by the receiver (high accuracy oscillator). Doppler effect (frequency change due to relative speed between the satellite and the receiver) and phase shift (small time shift between the waves) are repeatedly measured (1–20 times per second). From this data, very small relative displacement between satellites and receiver can be tracked. However, there is a large ambiguity on the total distance (number of integer wave cycles). The solving of these ambiguities – i.e. to find the real number of wave cycles between each satellite and the receiver – is the major issue of RTK. However, by using code data and redundant information from at least 5 satellites, it is possible to lock position. In this case, the theoretical accuracy (given by the manufacturers) of each position computation is between 0.5 to 2 cm horizontal and 1 to 3 cm vertical (with a small baseline, i.e. the short distance between base and rover receivers). This method is very sensitive to sudden satellite loss due to obstructions (missing epochs). Actually, a new ambiguity solving process may be needed each time that there is missing data in the phase and Doppler measurements. Like DGPS, RTK can be performed in real-time or in post processing.

## Validation of high accuracy GPS for gait analysis

Most applications of high-end GPS receivers in RTK-mode are static, i.e. implying the precise positioning of a fixed point on earth. Several studies report milimetric accuracy in this case [44], because it is possible to repeatedly measure the fix point and then calculate an average position with a greatly reduced error. Few applications need the kinematic use of RTK mode, i.e. the determination of a trajectory by repeatedly measuring a moving point with a high sampling frequency (10–20 Hz): therefore there are few validation studies in this research area.

In the field of wind engineering and industrial aerodynamics, Tamura and colleagues [45] recently demonstrated that GPS (RTK mode) was capable of an accurate assessment of small sinusoidal displacements (4–10 cm) in the 2–5 Hz frequency range by using a direct comparison with an electronic exciter. The sine-wave was correctly assessed, in terms of both amplitude and phase: the control and GPS curves were totally superimposed. In addition, 0.5 cm oscillation – an amplitude below the theoretical accuracy limits of GPS in RTK mode – was correctly tracked in terms of phase, but with small drift in amplitude in the +/- 1 cm range.

## High accuracy GPS: usability and practicability

Strict quality standards are needed in order to reach the highest possible accuracy with GPS in RTK mode for analyzing walking biomechanics: 1) the use of high-quality professional GPS receivers tracking both L1-L2 frequencies is required, such as *Topcon Javad *or *Leica*. 2) The time of the measurement must be carefully selected: additional satellites above 5, add redundant information that increases accuracy. We found that optimal accuracy was obtained with at least 7 GPS satellites. 3) No satellite below 20 degrees of elevation above the horizon must be used to reduce multipath (fake satellite signals induced by unpredictable reflections). 4) The smallest possible baseline for the best atmospheric error reduction is mandatory (500 m maximum between the reference receiver and the moving receiver). 5) Special attention should be paid during the RTK post-processing of raw GPS data: the missing epochs, cycle slips and unsolved ambiguities must be carefully monitored and the whole trial should be rejected if too many errors are found: in practice one out of five trial may be subjected to voluntary rejection.

Under such experimental conditions, we assumed that the theoretical limit of 1 cm accuracy could be reached and even overcome: it became possible to calculate gait parameters stride-by-stride. The main drawback is that optimal satellite constellation occurs infrequently during the day (i.e. typically 2 to 3 hours window in the diurnal period). In addition, similar weather conditions should be a pre-requisite to standardize the experiment (this is the case for every outdoor experiment). As a result, it is not possible to efficiently measure a large group of individuals with the current GPS technology.

In practice, our lab uses GPS/GLONASS receivers (Legacy E GDD, *Javad Navigation Systems*, San Jose, CA, USA). These devices can simultaneously track both American (GPS) and Russian (GLONASS) positioning system, increasing the total number of satellites available. The rover receiver and its power supply (total weight: 0.9 kg) are put into a backpack worn by the subject; the flat antenna (weight: 0.33 kg, 14 × 14 × 3 cm) is rigidly fixed onto a cap. The receivers can acquire both code and carrier phase up to 20 times each second (20 Hz). The raw data are post-processed by using the *Javad *Pinnacle software and its kinematic engine: the subject's trajectory is assessed by the double-difference method after phase ambiguity resolution. The 3D positions are converted into the Swiss grid coordinate system which provides distance measurements in metric units. The 3D speed vector was also computed for each point of the trajectory. In short, the output file of the trajectory processing contains seven columns for each epoch: time of the measurement (20 Hz, GPS time, nanosecond accuracy), North, East, Altitude (m), Speed North, Speed East, Speed altitude (m/s).

## From GPS positioning to gait parameters: the biomechanical assumptions

How can an antenna attached onto the top of the subject's head provide useful information about the stride by stride gait parameters? Beyond the question of positioning accuracy, 4 assumptions must be stated.

**Figure 3:**
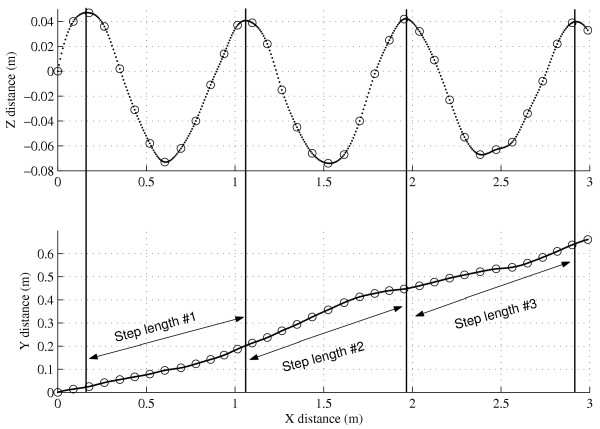
*Raw GPS data and measurement of the length of step*. One participant freely walked on the level ground. High precision GPS measured 3D positions of the moving participant with a centimeter accuracy at 20 Hz sampling rate (antenna fixed onto the head). The figure presents a small sample (3 m) of a 45 min. test. The top panel shows the sinusoidal variation of the vertical position (Z) as a function of the West-East (X) displacement. The bottom panel shows the South-North (Y) displacement as a function of West-East (X) displacement. The vertical lines indicate the beginning of each step. Dotted circles are raw 20 Hz GPS data. Small dots are 240 Hz interpolated positions.

1) *Average speed of the head over one gait cycle (two steps) is equal to the average body speed and hence average Walking Speed (WS)*. The head undergoes small rotations in different planes while walking [46]. However, there is no doubt that *on average *its speed is similar to the trunk and Center of Mass speed, because all body segments are interdependent. Therefore, the vector magnitude of 3D GPS speed vector can be averaged over one gait cycle to assess average walking speed.

2) *The head vertically oscillates at the same frequency as the trunk and Center of Mass: the frequency of this oscillation can be defined as Step Frequency (SF)*. The vertical oscillation of the head has been found to oscillate at the same frequency as the trunk [46]. We have also observed that average SF measured by GPS was identical to average SF measured by an accelerometer attached to the low back [47]. We agree that the definition of SF based on the head trajectory may be different than others, such as the inverse of stride duration, i.e. the time between to heel strikes measured by force plate or footswitch. However, in our opinion, different body segment can be alternatively used to track the rhythmicity of walking with comparable efficiency.

3) *One gait parameter can be computed by knowing the two others by the simple equation WS = SF × SL*. Because of the repetitive pattern of walking, WS, SF and SL are strictly related. Indeed, walking can be seen as iterative gait cycles in both spatial and temporal dimensions. To the temporal repetition after one stride duration, it adds a spatial repetition after one stride length. The rate at which the spatial repetition occurs is precisely the speed (distance/duration). In practice, the length of step can obviously be defined as the distance traveled by the head over one gait cycle. However, an alternative rationale is that there is no need to measure the 3 gait parameters: it is sufficient to measure two of them and deduce the third. SL can be therefore defined as the ratio between WS and SF. Alternatively, SF can be computed from SL and WS (SF = WS/SL).

4) *Accurate head trajectory can be assessed with a low sampling rate (10–20 Hz)*. The accurate assessment of head trajectory is the main requirement that make possible the computation of all gait parameters with GPS method. Indeed, the assumptions we have defined above (1–3) imply the recognition of a repetitive pattern in the raw trajectory signal in order to analyze each stride separately. In other words, the periodic return of a body segment to a similar state can be used to frame each gait cycle and hence to allow the measurement of the gait parameters stride by stride: the classical example is the repetition of heel strikes. In practice, we arbitrarily chose to detect the max altitude (peak) reached by the head on the vertical axis to define the beginning of each step (see fig. 3). The main obstacle to the detection of this point is that the head trajectory is not continuously tracked, but measured by the GPS receiver as successive discrete positions with a sampling rate ranging from 5 Hz [47-49] to 20 Hz [22]. We are convinced that such a sampling rate is sufficient to mathematically reconstruct the head trajectory with the required accuracy by interpolating extra-points between the GPS measurements. Indeed, there is a high correlation between successive points in the head trajectory, because of the inherent inertia and the low acceleration that are allowed by the system: a smooth trajectory is therefore expected. If the head would undergo small "erratic" unpredictable movements between two GPS points (1/20 s), this would imply a significant acceleration to the head (several g), and this is obviously not the case. In addition, multiple results in the literature clearly demonstrate that the body Center of Mass [24], the trunk [4], and the head [46] follow a sine-like, smooth, trajectory: the frequency of this sine-wave is precisely SF. From a digital signal processing point of view, it is obvious that a 10/20 Hz sampling rate is sufficient to perfectly describe a 1.5–2.5 Hz "sine-like" wave because of the Shannon's theorem. Fig. 3 illustrates the result of the interpolation process (spline interpolation) we apply to increase the temporal accuracy of head trajectory.

## High accuracy GPS and gait variability: the Lausanne results

In 1999 – in the field of physical activity assessment – we studied whether the combination of accelerometer with altimetry would lead to a major improvement of walking speed prediction in a variable slope environment [48]. The high accuracy RTK GPS with 5 Hz sampling rate was used as reference for speed and altitude measurement ("golden standard"). Because the trajectory assessment seemed very accurate, we tested the same instrument (Leica RTK GPS, 5 Hz sampling rate) to measure average walking parameters (WS, SL, SF) over 5 minutes steady state walking [47]. In addition, we measured vertical displacement and speed change stride-by-stride. We found that the average step duration measured with a portable accelerometer was statistically identical to GPS measurement. However, the parameters assessed stride by stride exhibited large variability. In a subsequent study, we attempted to assess average external power of walking [49]. However, the results were not totally in accordance with the results found in the literature, probably because of a poor recording of the phase shift between energy components [49]. More recently, we used a new device (10 Hz sampling rate) that allowed the recording of the basic gait parameters (walking speed, cadence, and step length) over several successive 5 sec periods [50]. We found that walking at low speed induced a different gait pattern compared to walking at preferred or high speed. In addition, slow walking exhibited higher variability of all gait parameters [50].

**Figure 4:**
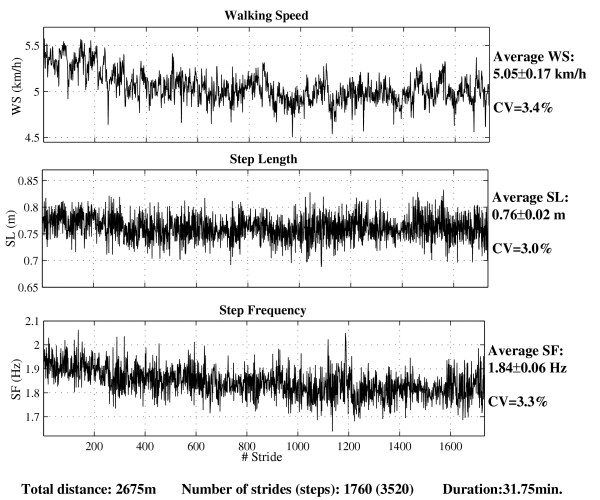
*Times series of gait parameters for a walking man (preferred speed)*. The gait parameters were measured in a male volunteer stride by stride (1 stride = 2 steps) over ~32 min. by using the high accuracy GPS method. The intra-individual (stride to stride) variability is expressed as both Standard Deviation (SD) and Coefficient of Variation (CV = SD/mean × 100). Total distance, number of strides and duration are indicated below.

The most recently study was conducted by applying the method explained above (20 Hz, strict standards) [22]. We analyzed gait parameters stride-by-stride in 8 subjects under free and constrained (metronome) conditions. We obtained time series as illustrated in fig. 4. This allows the analysis of the fluctuation of the gait parameters (walking speed, cadence, and step length) both in terms of amplitude (Standard Deviation, Coefficent of Variation) and dynamics (long range correlation, fractal pattern). Under free walking conditions, DFA (Detrended Fluctuation Analysis [20,21,51-53]) and surrogate data tests showed that the fluctuation of WS, SL and SF exhibited a fractal pattern (i.e., scaling exponent α: 0.5 < α < 1) in a large majority of participants (7/8). Under constrained conditions (metronome), SF fluctuations became significantly anti-correlated (α < 0.5) in all participants. However, the scaling exponent of SL and WS was not modified. We conclude that, when the walking pace is controlled by an auditory signal, the feedback loop between the planned movement (at supraspinal level) and the sensory inputs induces a continual shifting of SF around the mean (persistent anti-correlation), but with no effect on the fluctuation dynamics of the other parameters (SL, WS) [22].

## Advantages and drawbacks of GPS as compared to other methods

GPS technique falls under the category of methods that provide a limited set of biomechanical parameters with an increased practicability, such as, for example, portable accelerometers. The introduction of such a method will not displace high accuracy methods used in the "gait laboratories". However, it can provide useful alternative in the field of gait variability analysis, provided that the potential user is aware of the different constraints. In this context, table 1 summarizes the advantages and drawbacks of GPS.

**Table 1:** Potential advantages and shortcomings of the Global Positioning System (GPS) technique used for gait analysis

**Advantages**	**Shortcomings**
Available anywhere on the earth in any weather conditions for outdoor measurements at no cost	High cost of professional equipment
Tri-dimensional positioning with centimeter accuracy (Real Time Kinematics, RTK mode)	Not fully validated for gait analysis yet
No space restriction: freedom in the path selection, including uphill/downhill locomotion.	Limited time windows (2–4 h per day)
Free living conditions, i.e close to real life	One body segment measured only (head): Because of mandatory constant satellite access, the antenna must not be obstructed by body parts.
Unlimited number of consecutive strides: limited only by the memory capacity of the receiver and the duration of the batteries.	Outdoor analysis: difficult to standardize environmental conditions (weather, terrain).
	Not fully miniaturized (cumbersome antenna).

Regarding the technical and organizational obstacles, it seems that the high-accuracy GPS technology is difficult to implement for biomedical applications. Some obstacles are inherent to satellite positioning technique (outdoor experiments, optimal satellite access). However, future developments will increase the usability of the technique. The receivers become smaller with a higher computation power: new 100 Hz GPS chips are already available. Concerning GPS satellites, a challenging modernization program will offer a third civilian frequency (L5) for better availability and accuracy. New additional Russian GLONASS satellites will be also launched in the next few years. The European GALILEO system is planned for the next decade: it will provide a third independent positioning system. Consequently, the accuracy, availability and usability of satellite positioning have a substantial potential for growth.

The development of GPS technique for gait analysis is still embryonic. When the investigators will realize the potential of this new technology, they may use it as a complementary tool to better track the gait parameters of human being in their own "natural" environment. Given the importance of intra-individual variability of these parameters, "exportation" of the laboratory to free-living conditions may be the unique solution to analyze them over prolonged periods of time.
